# Efficacy of red ointment in wound cavity repair following non-puerperal mastitis debridement

**DOI:** 10.1186/s41065-025-00451-2

**Published:** 2025-05-19

**Authors:** Jiamei Feng, Zheng Chen, Jiaye Sun, Shijun Shao, Lu Xie, Wenchao Qu, Qingqian Gao, Xueqing Wu, Hua Wan

**Affiliations:** 1 Department of Mammary, Shuguang Hospital, Shanghai University of Tradition Chinese Medicine, No.185 Pu ’an Road, Huangpu District, Shanghai, 200011 China; 2 Department of Traditional Chinese Medicine, Yichuan Street Community Health Service Center, Putuo District, Shanghai, 200065 China

**Keywords:** Efficacy, Non-puerperal mastitis, Red ointment, Wound cavity repair

## Abstract

**Background:**

The objective of this study is to evaluate the efficacy of red ointment, a widely used topical agent in traditional Chinese medicine, in promoting wound cavity repair following debridement for non-puerperal mastitis (NPM).

**Methods:**

A prospective, randomized controlled trial was conducted, including 88 patients diagnosed with NPM. Patients were randomly assigned to either the treatment group or the control group. All patients underwent debridement during the acute inflammatory phase. Postoperatively, the treatment group received daily dressing changes using sterile gauze strips infused with red ointment, whereas the control group received sterile gauze strips soaked in rivanol. The effectiveness of treatment was assessed after two weeks by evaluating the total effective rate, wound cavity score, symptom and sign score, laboratory parameters, and adverse events.

**Results:**

In the intention to treat analysis, the total effective rate was 90.9% in the red ointment group, which was higher than the 86.4% observed in the rivanol group. In the per protocol analysis, the total effective rate was 97.6% in the red ointment group, exceeding the 92.7% in the rivanol group. Compared with rivanol-treated gauze strips, the use of red ointment gauze strips resulted in a significantly greater reduction in wound cavity volume (*p* < 0.05), improved local breast symptoms (*p* < 0.05), and a lower wound cavity score (*p* < 0.001). Granulation tissue in the red ointment group exhibited a significantly fresher color compared to the rivanol group (*p* < 0.05). No significant differences were observed between the two groups regarding adverse effects on hepatic and renal function following treatment.

**Conclusion:**

The use of red ointment gauze strips for wound cavity filling following NPM debridement demonstrated favorable clinical efficacy and safety, providing a viable option for postoperative drainage management.

## Background

Non-puerperal mastitis (NPM) is a chronic, refractory, aseptic suppurative inflammatory disease of the breast, characterized by a high recurrence rate [1]. It predominantly develops during the non-puerperal period and initially presents as a sudden onset of breast lumps. Over time, these lumps may undergo suppuration, leading to the formation of fistulas upon rupture through the skin, thereby increasing the likelihood of recurrence [2]. The prevalence of NPM accounts for approximately 4–5% of benign breast diseases, with an upward trend observed in recent years [3]. 

Surgical intervention remains a primary treatment modality for NPM [4, 5]. Among the various surgical approaches, acute phase debridement is the most frequently used. However, following debridement, the presence of pus and necrotic tissue often contributes to local inflammation and delayed wound healing due to the open nature of the wound cavity. Consequently, effective postoperative drainage is essential to minimize the accumulation of necrotic tissue and purulent material while facilitating granulation tissue formation, which is particularly crucial for wound cavity repair following NPM surgery.

Red ointment is a widely used topical agent in traditional Chinese medicine (TCM) surgery, primarily for its role in debridement and granulation tissue promotion. Prior studies have highlighted that red ointment exhibits anti-inflammatory properties, reduces edema and exudation, mitigates infection risk, and alleviates wound-related pain [6]. Currently, it is extensively applied in the management of chronic wounds, including pressure ulcers, diabetic foot ulcers, and other persistent skin ulcerations, as well as in wound healing following procedures such as anal carbuncles or fistula surgery [6, 7]. 

Currently, there is limited research on the clinical application of red ointment for wound management following NPM surgery. To assess its efficacy and safety in promoting wound healing, this study investigated red ointment, a widely used agent in TCM surgery known for its debriding properties and ability to facilitate granulation tissue formation. A comparative analysis was conducted to evaluate wound cavity repair outcomes following acute phase debridement in patients with NPM. The study assessed efficacy scores, symptom and sign scores, and laboratory parameters between the experimental group, which received daily dressing changes with red ointment gauze strips, and the control group, which received daily dressing changes with rivanol (thacridine lactate solution) gauze strips. The objective of these findings was to establish a theoretical foundation for the clinical application of red ointment in postoperative wound management for NPM.

## Clinical data

### Patients

Patients diagnosed with NPM who were hospitalized and underwent mastitis debridement in the Galactophore Department of Shuguang Hospital, affiliated with Shanghai University of Traditional Chinese Medicine, between April 1, 2023, and December 31, 2023, were included in the study.

### Inclusion criteria

1. Female patients meeting the diagnostic criteria for NPM [8].
2. Patients who underwent acute phase debridement in the hospital, with an open wound cavity left unsutured postoperatively, and who received postoperative management including fluid infusion and oral administration of TCM.
3. Patients who voluntarily agreed to participate and provided signed informed consent.

### Exclusion criteria

1. Pregnant and lactating women.
2. Patients with severe systemic diseases, severe mental disorders, or a recent history of significant trauma with incomplete recovery.
3. Patients diagnosed with severe malignant diseases.
4. Patients with a known allergy to the components of the investigational drugs, including red ointment and rivanol.

### Rejection criteria

1. Patients who were inadvertently included despite not meeting the inclusion criteria.
2. Patients who met the inclusion criteria but deviated from the trial protocol during treatment by using other topical or oral medications.
3. Patients who violated the study protocol significantly and did not undergo the designated treatment or assessments.
4. Patients who experienced severe allergic reactions to red ointment or rivanol during treatment.

### Drop-out criteria

1. Patients who were lost to follow-up.
2. Patients who withdrew from the clinical trial due to adverse reactions, perceived lack of efficacy, intolerance, or unavoidable physiological changes such as pregnancy or abortion during the study period.
3. Patients whose participation introduced significant bias in the clinical trial due to the use of other topical agents in the wound cavity or poor adherence to standard postoperative treatment protocols.

## Methods

### Sample size Estimation and random method

#### Sample size Estimation

The primary endpoint of this trial was the comparison of the total effective rate of open wound cavity repair following acute phase debridement for NPM between the red ointment group and the rivanol group. The sample size was determined using the “two-sample frequency comparison” method. Assuming an approximately equal distribution of participants between the two groups, the required sample size for each group was calculated using the following formula:

$$\:n=\frac{{\left({u}_{\alpha\:/2}+{u}_{\beta\:}\right)}^{2}}{2\times\:{\left(arcsin\sqrt{{P}_{2}}-arcsin\sqrt{{P}_{1}}\right)}^{2}}$$

Based on previous studies, it was assumed that the total effective rate of treatment would be approximately 0.6 in the rivanol group and 0.9 in the red ointment group [9, 10]. With a significance level (α) of 0.05 and a statistical power of 90%, an equal number of participants were allocated to each group. The minimum required sample size was calculated to be 40 per group, resulting in a total of at least 80 participants. Considering an anticipated dropout rate of 10%, the final sample size was determined to be 88, with 44 participants assigned to the red ointment group and 44 to the rivanol group.

#### Random method

Following the randomized block design scheme, four patients with closely aligned visit times were formed as block units. Within each block, patients were randomly assigned to different treatment methods to ensure balanced allocation across the study groups.

### General data

1. Age of onset: The mean age of onset was calculated, and the distribution of patients across different age groups was analyzed, with a five-year interval used for classification.
2. Body mass index (BMI): Height and weight measurements obtained on the day before treatment were used to calculate BMI. Participants were categorized based on standard BMI classifications: [11] underweight BMI < 18.5 kg/m^2^), the normal weight group (18.5 kg/m^2^ ≤ BMI < 24 kg/m^2^), the overweight group (24 kg/m^2^ ≤ BMI ≤ 28 kg/m^2^), and the obesity group (BMI > 28 kg/m^2^).
3. Previous medical history: The prevalence of other breast conditions, including acute mastitis, NPM, and benign breast tumors, was recorded. Additionally, the history of breast surgery, the occurrence of other medical conditions (e.g., pituitary microadenoma, mental disorders, thyroid disease), and medication use were documented.
4. Marital and reproductive history: The number of live births and abortions, the interval between the last birth or abortion and disease onset, and the distribution of patients based on these factors were analyzed.
5. Lactation history: The lactation history of patients with prior pregnancies was recorded.

### Incidence

This includes: the duration of disease, affected site, history of trauma (within 2 weeks of onset), maximum preoperative lump diameter (the maximum diameter of the largest lump was measured in case of multiple lumps) and treatment history.

### Therapeutic protocol and course of treatment

This is a non-blinded study in which different drainage gauze strips were randomly assigned to all patients on the first postoperative day.

### Red ointment group

Following disinfection of the breast surface, the wound cavity was packed with sterile gauze strips infused with red ointment. Red ointment, composed of 90% Vaseline, 10% calcined gypsum, and red litmus (Among them, red cinnabar is usually composed of substances containing mercuric oxide and mercuric nitrate), was prepared and supplied by the Drug Manufacturing Room of Shuguang Hospital, affiliated with Shanghai University of Traditional Chinese Medicine. The wound was then covered with sterile gauze, with an additional outer layer of gauze and a cotton pad of appropriate thickness to prevent exudation. The dressing was changed once daily.

### Rivanol group

After disinfecting the breast surface, the wound cavity was packed with sterile gauze strips soaked in Ethacridine Lactate Solution (manufactured by Guangdong Hengjian Pharmaceutical Co., Ltd., registration number GYZZ H44024244, 50 mL per bottle). The wound was then covered with sterile gauze, with an outer layer of gauze and a cotton pad of appropriate thickness to prevent exudation. The dressing was changed once daily.

Postoperatively, all patients received routine intravenous infusion of 0.75 g of Cefuroxime Sodium for three days to prevent infection. Additionally, conventional traditional prescriptions were uniformly administered as part of postoperative management.

### Observation time window

Day 0 (Day of surgery): On the day of the procedure, the wound cavity volume within the breast in its natural state was measured using the membrane adherence method following debridement surgery. Additionally, the wound cavity volume healing score was recorded.

Day 3 of treatment: A bacterial culture of the wound cavity was performed to assess microbial colonization and infection status.

Day 14 of treatment: Systemic and local symptom and sign scores were documented, along with assessments of hepatic and renal function. The wound cavity volume was remeasured, and the wound cavity volume healing score was reassessed to evaluate the progress of wound healing.

### Primary endpoint

The total effective rate of wound cavity repair was assessed based on changes in the wound cavity score. Clinical efficacy was classified according to the following criteria:

Cured: The wound cavity score decreased by ≥ 90%.

Significantly effective: The wound cavity score decreased by ≥ 60% and < 90%.

Effective: The wound cavity score decreased by ≥ 30% and < 60%.

Ineffective: The wound cavity score decreased by < 30%.

Total effective rate = [(number of cured patients + number of significantly effective patients + number of effective patients)/total number of patients] × 100%.

A comparison of the total effective rate between the red ointment group and the rivanol group was conducted using both per-protocol (PP) analysis and intention-to-treat (ITT) analysis [12]. The ITT analysis included data from all randomized patients, with those lost to follow-up or withdrawn from the study being considered treatment failures in the efficacy evaluation. The PP analysis excluded patients who withdrew or were lost to follow-up.

### Secondary endpoints

Wound cavity repair score after acute phase debridement: The evaluation of wound cavity repair included assessments of granulation tissue color, exudation, secretion characteristics, pain intensity, skin color and temperature, and wound cavity volume. Based on previous studies, the Non-Puerperal Mastitis Wound Cavity Efficacy Score Scale (hereafter referred to as the wound cavity score) was developed, as detailed in Annex A [13]. Granulation tissue color, exudation, secretion characteristics, and skin color and temperature were independently evaluated by two physicians at or above the attending level. Wound cavity volume was measured using the membrane adherence method. Pain intensity was assessed using the numeric rating scale. A lower total wound cavity score indicated a more favorable wound healing outcome.

Local and systemic symptom and sign score of NPM: To quantitatively assess disease severity and treatment response, two scoring systems were developed: The Systemic Symptom and Sign Score Scale of Non-Puerperal Mastitis and The Local Symptom and Sign Score Scale of Non-Puerperal Mastitis. These scales were based on the diagnostic criteria, common local and systemic manifestations of NPM, and their correlation with disease severity. The details of these scoring systems are provided in Annexes B and C. The severity and progression of NPM in different groups before and after treatment were systematically analyzed.

Bacterial culture: On Day 0 of treatment, a pus culture was obtained as the baseline, and the results were recorded. If bacterial growth was detected, a repeat culture of wound cavity exudate was conducted three days after the initial gauze strip change, following the original therapeutic protocol, to assess bacterial persistence or clearance.

### Safety indicators

Blood levels of alanine aminotransferase (ALT) and aspartate aminotransferase (AST) were assessed to evaluate hepatic function, while creatinine (Cr) levels were measured to assess renal function. These parameters were tested both before and after treatment. Test results exceeding 15% of the normal reference value were classified as abnormal.

### Statistical methods

Statistical analysis was conducted using SPSS 21.0. Measurement data following a normal distribution and demonstrating homogeneity of variance were analyzed using the two-independent sample t-test. Data that did not follow a normal distribution or exhibited heterogeneity of variance were analyzed using the Mann-Whitney U rank sum test.

Categorical variables were compared using Pearson’s chi-squared test or Fisher’s exact test, as appropriate. A value of *p* < 0.05 was considered statistically significant.

## Results

### General data

Between April 2023 and December 2023, a total of 88 patients meeting the enrollment criteria were included in the study, with 44 assigned to the red ointment group and 44 to the rivanol group. Six patients withdrew from the study due to poor clinical compliance. These participants were excluded from the PP analysis but were classified as treatment failures in the ITT analysis.

#### Age of onset

The average age in the red ointment group was 34.4 ± 5.9 years (range: 17–49 years), while in the rivanol group, it was 34.5 ± 8.4 years (range: 19–69 years). No statistically significant difference in age was observed between the two groups (*t* = 0.058, *p* = 0.663).

Age stratification was conducted in five-year intervals, beginning at age 15, and no significant difference was found in the distribution of age at onset between the two groups (*X*^2^ = 6.453, *p* = 0.488) (Table 1).

**Table 1:** Comparison of age stratified distribution

Age stratification (year)	Group		Total [n(%)]	X^2^	*P*
	Rivanol group [n (%)]	Red ointment group [n (%)]			
15–20	1(2.3)	1(2.3)	2(2.3)	6.543	0.488
21–25	1(2.3)	1(2.3)	2(2.3)		
26–30	9(20.5)	5(11.4)	14(15.9)		
31–35	20(45.5)	21(47.7)	41(46.6)		
36–40	9(20.5)	9(20.5)	18(20.5)		
41–45	2(4.5)	5(11.4)	7(8)		
46–50	0(0)	2(4.5)	2(2.3)		
51–55	0(0)	0(0)	0(0)		
56–60	0(0)	0(0)	0(0)		
61–65	0(0)	0(0)	0(0)		
66–70	2(4.5)	0(2)	2(2.3)		

#### BMI

The mean BMI in the red ointment group was 24.1 ± 4.4 kg/m² (range: 16.5–39.2 kg/m²), while in the rivanol group, it was 24.7 ± 4.3 kg/m² (range: 17.9–37.9 kg/m²). No statistically significant difference in BMI was observed between the two groups (*t* = 0.256, *p* = 0.614).

When stratified based on BMI categories, no significant difference in BMI distribution was found between the two groups (*X*^2^ = 2.128, *p* = 0.574) (Table 2).

**Table 2:** Comparison of BMI stratification

Group	*N* (case)	BMI stratification				X^2^	*P*
		Underweight [*n* (%)]	Normal weight [*n* (%)]	Overweight [*n* (%)]	Obesity [*n* (%)]		
Rivanol group	44	1(2.3)	21(47.7)	13(29.5)	9(20.5)	2.128	0.574
Red ointment group	44	4(9. 1)	22(50)	11(25)	7(15.9)		
Total	88	5(5.7)	43(48.9)	24(27.3)	16(18.2)		

#### Fertility

The median number of births was 1 in both groups. No statistically significant differences were observed in the number and distribution of births and abortions between the rivanol and red ointment groups (number of births: Z = -0.365, *p* = 0.715; number of abortions: Z = -1.708, *p* = 0.088).

The maximum duration between the last birth or abortion and disease onset was 43 years in the rivanol group, with a median duration of 4 years, while in the red ointment group, the maximum duration was 20 years, with a median duration of 4 years. No significant difference was found in the distribution of the duration between the last birth or abortion and disease onset between the two groups (Z = -0.678, *p* = 0.498).

#### Lactation history

A total of 38 patients in the rivanol group and 37 patients in the red ointment group had a history of lactation. No significant difference in the distribution of lactation history was observed between the two groups (*X*^2^ = 0.09, *p* = 0.764).

The median lactation duration was 11 months in both groups. The maximum lactation duration was 24 months in the rivanol group and 22 months in the red ointment group. No significant difference in lactation duration was found between the two groups (Z = -0.369, *p* = 0.712).

A history of puerperal mastitis was reported in 13 patients (34.2%) in the rivanol group and 15 patients (40.5%) in the red ointment group. No significant difference in the history of puerperal mastitis was observed between the groups (*X*^2^ = 0.321, *p* = 0.637).

#### Previous histories of breast diseases and other diseases

As presented in Table 3, no statistically significant differences were observed between the two groups in the comparison of previous breast diseases and other comorbid conditions (*p* > 0.05).

**Table 3:** Comparison of previous history

Previous history	Group		Total [*n*(%)]	X^2^	*P*
	Rivanol group [*n* (%)]	Red ointment group [*n* (%)]			
Breast benign tumor	4(9. 1)	4(9. 1)	8(9. 1)	0	1
Non-puerperal mastitis	8(18.2)	9(20.5)	17(19.3)	0.071	1
Pituitary microadenoma	2(4.5)	2(4.5)	4(4.5)	0	1
Mental disorders	2(4.5)	0(0)	2(2.3)	2.047	0.494
Other	4(9. 1)	4(9. 1)	8(9. 1)	0	1
Total	20(45.4)	19(43.2)	39(44.3)		

### Incidence

#### Diseased site

The preoperative distribution of the affected breast among the 88 patients was analyzed. The disease was located in the left breast in 55 patients (62.5%), in the right breast in 32 patients (36.4%), and in both breasts in 1 individual (1.1%) at the time of clinical evaluation. No statistically significant difference in the distribution of the affected site was observed between the two groups (*X*^2^ = 1.093, *p* = 0.825).

#### Trauma history

A history of trauma was reported in 5 patients (11.4%) in the rivanol group and 3 patients (6.8%) in the red ointment group. No statistically significant difference in trauma history was observed between the two groups (*X*^2^ = 0.550, *p* = 0.713).

#### Course of disease

The median disease duration before surgery was 2 months in both groups. The maximum preoperative disease duration was 10 months in the rivanol group and 5 months in the red ointment group. No statistically significant difference was observed in the preoperative disease duration between the two groups (Z = -1.272, *p* = 0.203).

#### Maximum lump diameter

Preoperative lump size was measured, and the mean maximum lump diameter was 9.8 ± 3.9 cm (range: 2–19 cm) in the rivanol group and 11.1 ± 3.9 cm (range: 3–18 cm) in the red ointment group. No statistically significant difference was observed in the preoperative maximum lump diameter between the two groups (*t* = -1.570, *p* = 0.120).

#### History of extramural hospital treatment

The comparison of extramural hospital treatment history between the two groups is presented in Table 4. No statistically significant difference was observed (*p* > 0.05).

**Table 4:** Comparison of history of extramural hospital treatment

History of extramural hospital treatment	Group		Total [*n* (%)]	X^2^	*P*
	Rivanol group [*n* (%)]	Red ointment group [*n* (%)]			
Aspiration biopsy	9(20.5)	16(36.4)	25(28.4)	2.738	0.098
Puncture pumping pus	9(20.5)	8(18.2)	17(19.3)	0.071	0.787
Antibiotics	18(40.9)	21(47.7)	39(44.3)	0.414	0.520
Hormones	12(27.3)	8(18.2)	20(22.7)	1.035	0.309
Chinese patent medicine	12(27.3)	8(18.2)	20(22.7)	1.035	0.309
Operation	3(6.8)	5(11.4)	8(9. 1)	0.550	0.713

#### Laboratory indicators

Blood lipid levels: Total cholesterol (TC) and triglyceride (TG) levels were analyzed in 88 patients using stratification. The results indicated that 24 patients exhibited marginal TC elevation, while 4 patients had elevated TC levels, collectively accounting for 41.8% of the total population. No statistically significant difference in TC levels was observed between the two groups (Z = -1.579, *p* = 0.114). Approximately 35.2% of patients exhibited marginal elevation to elevation in TG levels, with no significant difference between the two groups.

Blood routine parameters: Preoperative white blood cell count, neutrophil percentage, C-reactive protein, and erythrocyte sedimentation rate were recorded for both groups. A comparison of baseline blood routine parameters indicated no statistically significant differences between the groups (Table 5).

**Table 5:** Comparison of the number of patients with elevated preoperative blood routine items

Inspection item	Group		Total [*n* (%)]	X^2^	*P*
	Rivanol group [*n* (%)]	Red ointment group [*n* (%)]			
WBC	20(48.7)	15(36.5)	35(42.6)	0.691	0.406
N%	22(53.6)	30(73. 1)	52(63.4)	3.231	0.072
CRP	19(46.3)	14(34. 1)	33(40.2)	1.248	0.264
ESR	34(82.9)	32(78.0)	68(82.9)	0.283	0.595

### Baseline

#### Wound cavity score

The preoperative median wound cavity score was 20 points (range: 13–25 points) in the rivanol group and 21 points (range: 17–28 points) in the red ointment group. No statistically significant difference in the preoperative wound cavity score was observed between the two groups (Z = -2.666, *p* = 0.108).

#### Wound cavity volume

The median preoperative wound cavity volume was 27.5 mL in the rivanol group and 30 mL in the red ointment group. No statistically significant difference was observed between the two groups, indicating comparability (Z = -0.539, *p* = 0.590).

#### Local symptom and sign score

The preoperative median local symptom and sign score was 18 points (range: 10–28 points) in the rivanol group and 17 points (range: 10–32 points) in the red ointment group. No statistically significant difference was observed between the two groups, indicating any comparability (Z = -0.425, *p* = 0.671).

### Primary endpoint

#### Total effective rate

In the ITT analysis, the total effective rate was 90.9% in the red ointment group, which was higher than the 86.4% observed in the rivanol group. In the PP analysis, the total effective rate in the red ointment group was 97.6%, exceeding the 92.7% recorded in the rivanol group. The comparative effectiveness analysis between the two groups is presented in Table 6.

**Table 6:** Comparison of clinical effects

Analysis Type	Group	*N* (case)	Cured [*n* (%)]	Significantly effective [*n* (%)]	Effective [*n* (%)]	Ineffective [*n* (%)]	Z	*P*
ITT analysis	Rivanol group	44	1(2.3)	5(11.4)	32(72.7)	6(13.6)	-4.472	< 0.001
	Red ointment group	44	5(11.4)	25(56.8)	10(22.7)	4(9.1)		
PP analysis	Rivanol group	41	1(2.4)	5(12.2)	32(78)	3(7.3)	-5.04	< 0.001
	Red ointment group	41	5(12.2)	25(61)	10(24.4)	1(4.9)		

### Secondary endpoints

#### Wound cavity score

The pre- and post-treatment wound cavity scores were 19.51 ± 3.28 and 10.26 ± 3.40 in the rivanol group, and 21.39 ± 2.33 and 6.51 ± 5.18 in the red ointment group, respectively. No significant difference was observed in the pre-treatment wound cavity score between the two groups (t = 0.230, *p* > 0.05). However, after treatment, the wound cavity scores in both groups significantly decreased compared to pre-treatment values *(p* < 0.001), with a more pronounced improvement in the red ointment group *(p* < 0.001).

##### Wound cavity volume reduction rate

ITT analysis: The wound cavity volume reduction rate was significantly higher in the red ointment group compared to the rivanol group (*t* = -3.808, *p* < 0.001). The median wound cavity volume reduction rate was 77.8% (0–91%) in the red ointment group and 49.3% (0–90%) in the rivanol group.

PP analysis: The wound cavity volume reduction rate remained significantly higher in the red ointment group than in the rivanol group (50% [15–90%] vs. 77.7% [33–91%], *t* = -5.298, *p* = 0.003, *p* < 0.05).

##### Pain score

The pre- and post-treatment pain scores were 4.26 ± 2.14 and 1.30 ± 1.60 in the rivanol group, and 4.95 ± 1.99 and 1.67 ± 1.84 in the red ointment group, respectively. After treatment, pain scores significantly decreased in both groups compared to pre-treatment values (*p* < 0.001). However, no significant difference was observed in the change in pain scores between the two groups (*p* = 0.362).

##### Granulation color score

The granulation color scores before and after treatment were 3.00 ± 1.03 and 1.35 ± 1.25 in the rivanol group, and 2.90 ± 1.00 and 1.09 ± 0.95 in the red ointment group, respectively. No significant difference was observed in pre-treatment granulation color scores between the two groups. After treatment, granulation color scores significantly decreased in both groups (*p* < 0.001), with a more pronounced improvement in the red ointment group (*p* = 0.046).

##### Wound cavity exudation score

The pre- and post-treatment exudation scores were 2.54 ± 0.70 and 1.30 ± 1.09 in the rivanol group, and 2.57 ± 0.87 and 1.85 ± 1.16 in the red ointment group, respectively. No significant difference was observed in pre-treatment exudation scores between the two groups. After treatment, exudation scores significantly decreased in both groups (*p* < 0.001), but the change in exudation scores before and after treatment did not differ significantly between the two groups (*p* = 0.116).

##### Secretion property score

The pre- and post-treatment secretion property scores were 3.04 ± 0.90 and 1.45 ± 1.04 in the rivanol group, and 3.12 ± 0.91 and 1.82 ± 1.21 in the red ointment group, respectively. No significant difference was observed in pre-treatment secretion property scores between the two groups. After treatment, secretion property scores significantly decreased in both groups (*p* < 0.001), but no significant difference was found in the change in secretion property scores before and after treatment between the two groups (*p* = 0.243).

##### Skin color and temperature score

The pre- and post-treatment skin color and temperature scores were 2.02 ± 1.11 and 0.83 ± 1.01 in the rivanol group, and 2.00 ± 0.84 and 1.12 ± 1.28 in the red ointment group, respectively. No significant difference was observed in pre-treatment skin color and temperature scores between the two groups. After treatment, skin color and temperature scores significantly decreased in both groups (*p* < 0.001), but the difference in pre- and post-treatment scores between the two groups was not statistically significant (*p* = 0.297).

#### Systemic symptom and sign score

The systemic symptom and sign scores before and after treatment were statistically analyzed for both groups. The results indicated a significant reduction in systemic symptom and sign scores following treatment (*p* < 0.001). However, no significant difference was observed between the two treatment groups in terms of their effect on systemic symptom and sign scores (*p* = 0.546) (Table 7).

**Table 7:** Comparison of systemic symptom and sign score

Group	*N* (case)	X ± S before treatment (point)	X ± S after treatment (point)	X ± S in d (before-after treatment) (point)	*P* Intra-group	*P* Inter-group
Rivanol group	41	0.45 ± 0.95	0.25 ± 0.15	0.42 ± 0.98	< 0.001	0.546
Red ointment group	41	0.73 ± 1.63	0.09 ± 0.48	0.64 ± 1.75	< 0.001	

#### Local symptom and sign score

The local symptom and sign scores of the breast before and after treatment were statistically analyzed for both groups. The results demonstrated a significant reduction in local symptom and sign scores following treatment (*p* < 0.001). Additionally, the improvement in local symptoms and signs was significantly greater in the red ointment group compared to the rivanol group (*p* = 0.043) (Table 8).

**Table 8:** Comparison of local symptom and sign score

Group	*N* (case)	X ± S before treatment (point)	X ± S after treatment (point)	X ± S in d (before-after treatment) (point)	*P* Intra-group	*P* Inter-group
Rivanol group	41	17.0 ± 4.44	2.76 ± 2.62	14.23 ± 4.58	< 0.001	0.043
Red ointment group	41	17. 8 ± 4.87	0. 8 ± 1.09	17.02 ± 4.76	< 0.001	

#### Bacterial culture

Among the 82 patients who completed the trial, bacterial cultures on day 1 of treatment were negative. However, wound cavity pus cultures on day 3 revealed bacterial infections in 11 patients, including 5 in the rivanol group (all infected with *Staphylococcus epidermidis*) and 6 in the red ointment group (4 infected with *S. epidermidis*, 1 with *S. lugdunensis*, and 1 with *Corynebacterium kroppenstedtii*). No statistically significant difference in bacterial infection rates was observed between the two groups based on the chi-square test.

### Safety evaluation

Among the 82 patients who completed the trial, six exhibited elevations in AST and ALT levels related to hepatic function following treatment. These elevations remained below twice the normal reference value. No abnormalities in Cr levels related to renal function were observed.

Of the six patients, four (9.5%) were in the rivanol group and two (4.8%) were in the red ointment group. No statistically significant difference was found in the incidence of hepatic or renal function-related adverse reactions between the two groups (χ² = 0.297, *p* = 1.000).

A follow-up hepatic and renal function assessment was conducted seven days after discontinuation of the treatment, indicating that all six patients had returned to normal renal function levels.

## Discussion

Within the first 14 days following NPM surgery, the wound cavity predominantly remains in the inflammatory response and granulation tissue proliferation stage. During this period, local inflammation may intensify, and wound healing may be delayed due to the large wound cavity volume, impeded cell migration, and inadequate drainage of fat necrotic tissue fluid and pus produced during the inflammatory phase. Red ointment is commonly applied to open wounds to facilitate necrotic tissue removal and granulation tissue formation.

Prior studies demonstrated the efficacy of red ointment in wound healing. Jiang et al. reported that perianal abscesses treated with red ointment-filled drainage wounds exhibited significantly shortened healing times and lower recurrence rates compared to the direct open wound group [6]. Similarly, Li et al. investigated the application of Qufu Shengji Powder and red ointment gauze strips for postoperative anal fistula wounds and observed a significant therapeutic effect, with improved secretion scores and superior granulation tissue color and morphology in the red ointment group compared to the control group [13]. Based on these findings, this study was conducted to evaluate the efficacy of red ointment in wound cavity repair following comedogenic acute mastitis surgery.

The findings of this study indicate that NPM primarily affects patients aged 26–40 years, with 89.7% having a history of pregnancy (median duration between the last birth or abortion and disease onset: 4 years) and 85.2% having a history of lactation (median lactation duration: 11 months). Among these, 37.3% developed puerperal mastitis during lactation, and 45.5% had a BMI in the overweight to obese range. These data indicate that NPM is more prevalent among multiparous patients of childbearing age with a history of lactation and a higher BMI.

Prior research on the risk factors for comedogenic acute mastitis identified autoimmune diseases, congenital nipple inversion, nipple trauma, oral contraceptive use, previous lactation disorders, overweight or obesity, breast trauma, insomnia, and anxiety as independent risk factors for the condition [4, 14]. These findings are consistent with the results of the present study. Additionally, more than 60% of cases involved the left breast, and 9.1% had a history of breast trauma within two weeks prior to disease onset. Prior studies confirmed that a history of trauma is also an independent risk factor for NPM [4]. A possible explanation is that trauma-induced breast duct rupture may stimulate ductal epithelial cells to produce lipophilic substances that leak out of the duct via the phospholipid bilayer, triggering an immune response involving immune cell activation. This process ultimately contributes to the formation of inflammatory nodules and the development of inflammation [15]. 

In this study, wound cavity dressings were changed using red ointment gauze strips and rivanol gauze strips following acute phase debridement for NPM. After 14 days of treatment, a significant reduction was observed in the wound cavity score, systemic symptom score, and local symptom and sign score in both groups. Both ITT analysis and PP analysis demonstrated that the total effective rate was significantly higher in the red ointment group compared to the rivanol group.

Analysis of wound cavity scores indicated that the red ointment group exhibited a greater improvement in granulation tissue color compared to the rivanol group. This indicates that wounds treated with red ointment gauze strips developed granulation tissue with a fresher color, reflecting the ability of red ointment to facilitate necrotic tissue removal and promote granulation tissue formation. A possible mechanism underlying this effect is that the small quantity of mercury ions present in red ointment may provide continuous and mild stimulation to the wound cavity, thereby enhancing local blood supply and nutritional support.

Additionally, the granulation color score, wound cavity volume reduction rate score, exudation property score, skin color and temperature score, and pain score significantly improved in both the red ointment and rivanol groups after treatment, indicating that adequate drainage following NPM debridement effectively contributes to local wound cavity repair. However, compared to the rivanol group, the red ointment group exhibited significantly fresher granulation tissue color and a greater reduction in wound cavity volume, further supporting the role of red ointment in promoting granulation tissue formation and necrotic tissue removal.

During the study, bacterial infections were observed in 11 patients during dressing changes, with *S. epidermidis* accounting for 81.8% of the isolated strains. No significant difference in bacterial infection rates was detected between the two groups. Additionally, one patient had *C. kroppenstedtii* in the pus culture results. This bacterium has been identified as being associated with a specific subtype of cystic neutrophilic granulomatous mastitis in NPM [16]. Further research is required to examine targeted therapeutic strategies for patients diagnosed with *C. kroppenstedtii*-related infections.

Among the 82 patients who completed the trial, six exhibited elevations in AST and ALT levels related to hepatic function following treatment, with all elevations remaining below twice the normal reference value. No abnormalities in Cr levels related to renal function were observed. Chi-squared analysis indicated no significant difference in the incidence of hepatic and renal function-related adverse reactions between the two groups.

The findings from this trial further reinforce that the continuous use of low-dose red ointment gauze strips for 14 days has minimal impact on hepatic and renal function.

Shengdan has been the “sacred medicine for surgery” that has been used for thousands of years. Through a large number of clinical and experimental studies, it has been verified to have good effects of promoting the discharge of pus, removing necrotic tissue, and promoting tissue regeneration. Shengdan has played a huge role in the treatment of inflammatory and difficult-to-heal wounds. Currently, the Jiu Yidan, which is a preparation of Hongshengdan most commonly used in clinical practice, is composed of calcined gypsum and Hongshengdan, with a ratio of 9:1. The main chemical components of Shengdan are HgO, Hg(NO_3_)_2_, etc. Mercury can be excreted through the kidneys, liver, and colonic mucosa, mainly through the kidneys, accounting for 75% of the total amount of absorbed mercury.

A clinical study has shown that when Jiu Yidan is applied externally continuously at a dose of 1.5 mg/cm² for 7 to 11 days during the treatment of acne mastitis, no acute or chronic mercury poisoning symptoms were observed in patients during the treatment process, the drug withdrawal observation period, or the follow-up period. However, according to the laboratory examination results, the blood and urine mercury levels after medication and one day after drug withdrawal were significantly higher than those before medication (*P* < 0.001). The blood mercury level returned to the normal range 14 days after drug withdrawal, and the urine mercury level returned to the normal range three months after drug withdrawal [17]. 

An animal experiment shows that the Hg level in rabbit blood and urine was significantly increased after the consecutive administration of double-dose Jiuyi Dan for 1 month. However, the blood Hg level and urine Hg level recover after the drug withdrawal for 71 days and 3 months, respectively. The liver and kidney indicators do not significantly change with the dose [18]. 

## Conclusion

The use of red ointment gauze strips for wound cavity drainage following NPM surgery has demonstrated favorable clinical efficacy and safety. Treatment with red ointment was associated with a significant reduction in wound cavity scores and an increase in the wound cavity volume reduction rate, thereby facilitating adequate blood supply for granulation tissue formation, effective necrotic tissue removal, and enhanced granulation growth. These findings indicate that red ointment is a viable option for clinical application in postoperative wound management.

## Data Availability

All data generated or analysed during this study are included in this article. Further enquiries can be directed to the corresponding author.

## Abbreviations

NPM: Non-puerperal Mastitis
BMI: Body Mass Index
WBC: White blood cell
N%: Neutrophil%
CRP: C-reactive protein
ESR: Erythrocyte Sedimentation Rate
PRL: Prolactin
